# An analytical performance approach for RCS/RS with one robot serving multiple stack heights under a one-path relocation strategy

**DOI:** 10.1038/s41598-024-53884-6

**Published:** 2024-02-13

**Authors:** Philipp Trost, Michael Eder

**Affiliations:** Institute for Engineering Design and Product Development, Technische Universität Wien, Lehargasse 6, Objekt 7, Hoftrakt BD, 1060 Vienna, Austria

**Keywords:** Automated warehouses, RCS/RS, Cycle time model, Multiple-deep storage stacks, Grid-based storage system, Engineering, Mechanical engineering

## Abstract

Robotic compact storage and retrieval systems (RCS/RS) represent a modern and useful storage system since the number of installed systems is growing fast. The modularity and demand-based scalability are reasons, therefore. Nonetheless, there are hardly any statements on the performance of those warehouses. This paper presents an analytical calculation approach to determine the performance of an RCS/RS with one operating robot serving different grid sizes and a varying number of stacked containers. The robot’s cycle time is calculated by assuming a uniform distribution of container stacks and a probabilistic storage height. A discrete-event simulation model of an RCS/RS is built to verify and validate the analytical approximations. The system’s basic structure and the input parameters originate from a European material handling provider. After the verification and validation, an extensive parameter variation is done with the target of displaying a wide range of usage. This analytical approach, which is easy and fast solvable with standard calculation programs, represents an easy and fast tool to predict the performance of one robot operating in an RCS/RS for any system configuration.

## Introduction

At the end of 2023, the robotic compact storage and retrieval system (RCS/RS) provider *AutoStore* announced the deployment of 50,000 robots worldwide, representing a significant milestone for the company. This illustrates the unbroken trend towards automated warehouses and highlights that the advantages of those systems are in demand. In addition, the number of companies providing RCS/R systems besides *AutoStore* is continually increasing.

**Figure 1:**
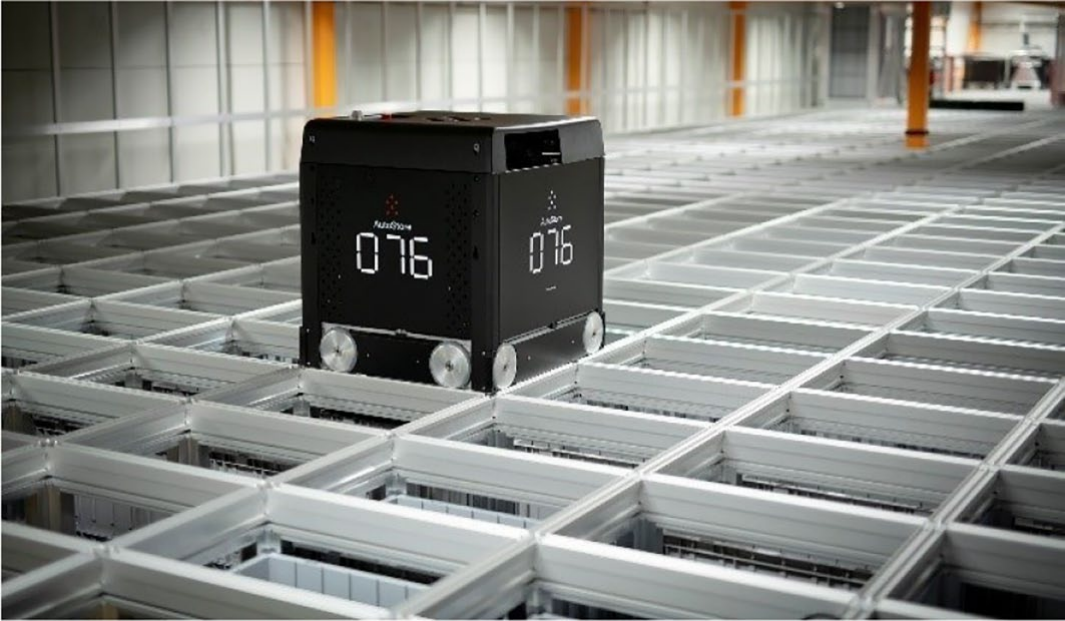
Robot on the grid. Source: AutoStore^1^.

From a technical point of view, such storage systems are reliable, fast, modular, scalable, efficient, and sustainable. RCS/RS use plastic containers stacked on each other and arranged in a block for storing the goods and robots (Fig. 1) for transporting the containers. I/O shafts with picking stations at the edges are used for in- and output. The number of robots, the number of picking stations, the number of containers, and hence the grid size can be expanded on the basis of demand.

The main difference to classical automatic storage and retrieval systems (AS/RS) are the grid instead of the storage rack, which also serves as a railway network for the robots. The elimination of the aisles due to the operation by the robots from above enables the highest storage densities. Compared to classical storage and retrieval machines (S/R machines), the robots are forced to separate their movements along the two horizontal axes. Additionally, RCS/RS usually have storage heights of up to 25 stacked containers^2^. This probably results in a high amount of relocations that are necessary before a container can be retrieved. The system’s complexity and the fact that there are hardly any general statements, neither scientific nor on the sales side, about throughput or system design reveal the need for scientific consideration.

This paper’s target is to develop an approach to calculate the throughput of an RCS/RS with one operating robot serving one picking station at one edge of the grid, considering an inhomogeneous article distribution. Because of the system’s complexity, extensive literature research will be done in order to gain insights into how the performance of similar systems, e.g. AS/RS or SBS/RS, can be predicted. Based on that, an analytical approach for RCS/RS will be presented, valid for storage heights from one up to a theoretically unlimited number of stacked containers. The number of stacks along the horizontal axes can also be varied from tiny to large systems. To check the accuracy of the analytical approach, a numerical simulation study using a discrete event simulation (DES) developed for this paper will provide results to compare.

Nowadays, material handling providers have to simulate nearly every new storage system to know the possible throughput for one specific configuration scenario, which is, in most cases, time and computationally-intensive. This analytical approach’s main advantage and novelty is the fast and straightforward method to determine the possible throughput of one robot operating in an RCS/RS for a given set of input parameters and system configurations. The equations can be easily implemented in a table calculation program or a parametric computer algebra system.

To give an overview of the paper, section "RCS/R system description" describes RCS/R systems in a more detailed way before section "Literature review" discusses the existing literature according to RCS/R systems as well as a short comparison with other, similar systems such as SBS/RS or puzzle-based storage systems (PBSS). Concerning section "Literature review", section "Analytical approach" presents the analytical performance calculation approach. A numerical study is done in section "Numerical study" to validate and verify the analytical approach as well as to make statements on how such systems behave and how the approach can be deployed. To close this paper, section "Conclusion and outlook" gives a summary and an outlook for further research.

## RCS/R system description

**Figure 2:**
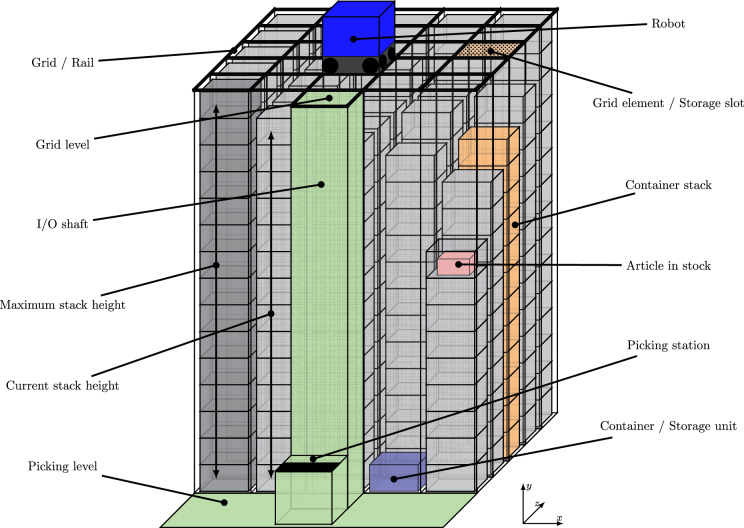
RCS/RS.

RCS/R systems are fully automatic, by robots from above-operated warehouses, that store small goods in standardised plastic containers stacked on each other using the Last-In-First-Out (LIFO) storage strategy within each stack. The storage and retrieval are done from the top, which leads to very high volume-density rates because of the loss of the aisles. Figure 2 exhibits a small section of an RCS/RS.

Ten Hompel et al.^3^ were, beside Wehking^4^, the first who mentioned RCS/R systems in a relevant logistical volume and gave an overview of the used technology and the advantages such as high efficiency, flexibility, and modularity. This can be expanded by high storage density, low space utilisation, high reliability due to high redundancy, and simple design (Kartnig et al.^5^).

Basically, RCS/R systems consist of five main components:

- Storage grid
- Containers
- Robot
- Picking station with I/O shaft
- Controller (connecting the system’s control with the warehouse management system)

The storage grid serves as an orthogonal railway network for the robots and as a divisional grid for the stacked storage containers. The goods to be stored inside the warehouse get put into plastic containers stacked on each other. Depending on the height of the containers and the load or the height of the hall, a maximum stacking height for the RCS/RS results. The third component is the autonomous vehicle, which carries out the storage and retrieval process. The battery-operated robot picks up the filled containers at the input and output shaft (I/O shaft). It transports them along the railway grid to the assigned grid element, where the container is lowered down onto the stack. The picking station is the fourth main component used as the warehouse’s input and output point (I/O point). On the one hand, the containers are filled with storage goods. On the other hand, the ordered containers get emptied by picking the required goods out of the containers to complete the customer’s order. The grid level and the picking station in front of the storage system are connected by the I/O shaft. The system investigated in this paper consists of an I/O shaft, where the robots self lift and lower the containers. A more detailed description of the modules of RCS/RS, the processes and a definition of the technical terminology can also be read in Trost et al.^6^.

RCS/R systems can be deployed in different operation modes: Single (SCC) or dual command cycle (DCC). According to^7^, separate storage and retrieval are called single command cycles. Dual command cycles combine storage and retrieval to reduce the number of empty runs. As mentioned above, some systems also return relocate, which represents another differentiation. Therefore, section "Cycle time calculations" gives a detailed description of all operation modes.

**Figure 3:**
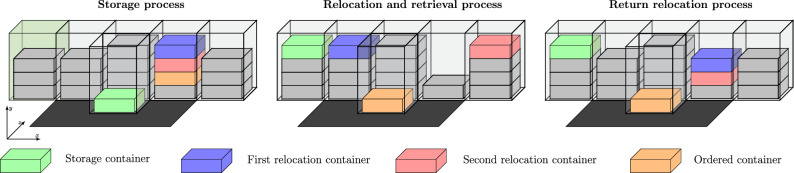
Visualised storage, relocation, retrieval process, and return relocation process.

To give an insight into the processing of RCS/RS, Fig. 3 shall illustrate the tasks. First, the green container at the picking station in the middle of the edge shall be stored on the green-marked stack. Therefore, a robot will be assigned to pick up the green container at the picking station, lift it through the I/O shaft, and transport it to the correct stack. The storage process is completed after lowering down the container onto the stack.

If a new order to retrieve without having direct access to the required container arrives, it is necessary to relocate all the other containers stacked above the required one. In the example of Fig. 3, the orange container is required and has to be retrieved. This can only be done by relocating the blue and the red containers stacked above the orange ones. The relocation containers are transported to neighboured stacks that provide space for another container. Based on Fig. 3, the blue container gets relocated to the stack on the left side next to where the storage was done before and the red container gets relocated to the far right stack. Only now the orange container can be retrieved. To ensure that relocations are always possible, the filling degree of the storage system is limited to a specific value (between 85 and 95%).

While some storage systems would remain at this stage and start a new storage or order, other storage systems, after the required container is retrieved, return relocate the prior relocated containers in the sequence of their removal. For the example in Fig. 3, this would mean that the red and the blue containers are return relocated to their initial stack. Since most of those systems carry out the return relocations immediately after the retrieval of the ordered container, the red container can then, after return relocation, be found where the orange container was initially. Analogous, the blue container is now again stacked above the red one.

In case of a distinctive class-based article distribution (e.g. ABC-article structure), it can be advantageous to return relocate all those relocated containers to their original stack. Storage systems with an inhomogeneous article structure do not return relocate since this would be just another throughput-decreasing factor. Whenever the articles from the retrieved container are taken, the orange container can be restored to the warehouse on top of any stack providing space. This does not necessarily mean to be the initial stack with the blue container.

## Literature review

The discussion of automatic storage and retrieval systems (AS/RS) in a scientific context started in the 70’s of the last century. The first systems, mostly AS/RS, were invented and installed, with big and heavy storage and retrieval machines (S/R machines). Therefore, analytical performance calculation approaches, so-called cycle time models (CTM), were developed to gain statements based on the kinematic data of the S/R machines and found their way into commercial standards. The advancement brought improved systems with, e.g. aisle-free S/R machines, load-handling devices for more than one tote, or multiple-deep storage racks.

Based on classical AS/RS and concomitant with the demand for higher order speed and higher space utilisation rates, three-dimensional AS/RS (3D-AS/RS) were invented at the beginning of this millennium. Those systems consist of an automatic crane that moves the totes horizontally and vertically. Hence, the free space for aisles is minimised, and the space utilisation rate is maximised since the totes can be moved and stored along the third direction. De Koster et al.^8^ were the first to investigate the performance and the optimal design of 3D-AS/RS with a random storage strategy for single command cycles (SCC) by developing a cycle time model. A gravity-supported or powered conveying mechanism does the depth transport of the storage units. Based on de Koster’s model^8^, Yu et al.^9,10^ did further studies using different storage strategies, while Yang et al.^11^ considered the acceleration/deceleration of the S/R-machine. Hao et al.^12^ varied the location of the I/O point, and Xu et al.^13^ developed travel time models for dual command cycle (DCC) and lower-mid dwell points.

Using little transport vehicles instead of belt conveyors or gravity slides leads to higher availability rates caused by higher redundancy. Such systems are commonly known as automatic vehicle storage and retrieval systems (AVS/RS) or shuttle-based storage and retrieval systems (SBS/RS). Zaerpour et al.^14^ investigated a 3D-AVS/RS with shuttle vehicles for horizontal transport and the consideration of a dedicated and shared storage policy. Azahdeh et al.^15^ developed an analytical model to predict the optimal layout of a vertical RCS/RS (V-RCS/RS), which has a layout similar to SBS/RS, and to analyse the performance considering two different robot blocking protocols with a closed queuing network. Both used numerical simulation and empirical data from a material handling provider for validation.

SBS/RS also have been a huge research topic since the variety of the systems is enormous. A literature review only within the last five years by Li et al.^16^ found 41 papers considering the framework topics of physical design, control strategy, and performance evaluation. Generally, a shuttle is installed for one tier within one aisle and transports the totes from/to the lift in front of each aisle (tier-captive, aisle-captive), enabling high throughput rates. Other systems use tier-free shuttles to reduce the number of shuttles required. Lately, some tier-free shuttles can serve multiple-deep racks and, in some cases, also multiple-high and multiple-deep storage systems. Eder^17^ presented a method for determining the performance of an SBS/RS working with vehicles along multiple tiers and/or multiple deep storage slots using a cycle time model to predict the shuttle’s interarrival time at the lift.

Puzzle-based storage systems are arranged in a block and use a lift for vertical and, unlike RCS/RS, load-captive shuttles for horizontal transport. Those systems, characterised by a very high storage density, were under the investigation of Gue^18,19^. He focused only on the location and arrangement of the storage units on the grid within one tier. Gue and Kim derived an expression for the retrieval within a 4 by 4 grid with 15 totes and only one empty slot close to the I/O point. Kota et al.^20^ expanded the approach for an empty slot located anywhere on the grid. Gue and Furmans^21^ developed the GridStore, representing a modular, scalable and decentralised high-density storage system. The authors depict that the performance underlies various operation configurations and can operate deadlock-free. Later on, Zaerpour et al.^22^ took multiple tiers into account and calculated the optimal system dimensions regarding a minimum retrieval time.

Based on the advantages of the systems mentioned above, RCS/RS were invented using independent vehicles (robots) on top of a block layout storage system with container stacks. The number of scientific works is still small. Azadeh et al.^23^ and de Koster^24^ presented developments and research opportunities in the field of robotised and automated warehouses. They found that many new warehouses and robotic systems, as well as RCS/RS, have hardly been studied.

Zou et al.^25^ were the first to publish an analytical approach for performance evaluation of RCS/RS using a semi-open queuing network (SOQN). This was done under the assumption of numerous simplifications and introducing a “wall parameter”. They explored chaotic and sorted warehouse strategies to gain the optimal length-to-weight ratio and stack height. Mutual hindrances of the robots were not further considered since the number of robots was small compared to the grid size. The central statement of the investigation was that the costs for the sorted warehousing (only one type of article in each stack)—which is atypical for RCS/RS—could be twice as high as with the chaotic strategy significantly since sorting would reduce the great advantage of the high degree of space utilisation. The sorted system has a significantly higher handling capacity since relocations are minimised or eliminated.

Galka et al.^26^ carried out a user study among 64 AutoStore-system users and provided general results on grid sizes in operation, the number of robots and picking stations used, shift models and ordered items per hour. Based on this, the authors formed ratios such as the number of robots per grid size or the number of stacks, the number of picking stations per grid size or the number of robots per picking station.

Trost et al.^6^, Beckschaefer et al.^27^, Ko et al.^28^, and Galka et al.^29^ all developed a discrete event simulation with specific system characteristics in order to gain statements about the system. While Beckschaefer et al.^27^ focused on warehousing strategies and whether a new product should be stored in an empty container or an already with the same product partially filled container should be removed from storage to store the new stock item, Ko et al.^28^ proposed a roll-out heuristic algorithm with the target of finding the optimal order sequencing within an RCS/RS.

Tjeerdsma^30^ developed a multi-scenario discrete event simulation to redesign an order-processing line for the Dutch post. Hameed et al.^31^ developed a numerical performance calculation approach using an optimal path algorithm for robot routing and compared the impact of a collision avoidance system within the robots. For one specific testing scenario, the total throughput decreased by around 10 percent with the consideration of obstacles compared to neglecting them. Chen et al.^32^ investigated overhead RCS/RS (ORCS/RS) with overhead cranes (“bridge crane”) by using dedicated and shared storage policies within the stacks and zoning within the warehouse by numerical discrete event simulation.

Since the review has shown that there is only one analytical performance determination approach using a SOQN, which is neither easy nor fast analytically solvable, this paper aims to present a straightforward tool to predict the throughput of an RCS/RS with one robot serving one picking station at one edge of the grid. The approach is based on Eder^33^ and enhances the CTM for RCS/RS with stack heights up to a theoretically unlimited number of stacked containers.

## Analytical approach

Section "Analytical approach" presents the analytical approach to calculate the throughput of an RCS/RS with one robot. This is an easy and fast tool to predict the system’s performance with many parameters that can be varied. To calculate the performance of a whole RCS/R system, knowing the cycle time of one robot is mandatory. This paper presents the CTM based on Eders^17^ SBS/RS performance approximation. While Eder^17^ uses an open-queuing model with limited capacity, this paper’s model is limited to calculating the cycle time of one robot on the grid to determine its throughput.

Based on the system description (section "RCS/R system description"), the main assumptions and some simplifications that have to be made for the analytical approach are listed below:

- The robot works in a single or dual command cycle under the First-Come-First-Served (FCFS) rule.
- The system’s dwell point is in front of the I/O shaft.
- The I/O shaft is located in the middle of one of the grid’s edges.
- There are always totes waiting at the dwell point in front of the I/O shaft.
- The robot’s velocity is constant. If not, a realistic velocity rate has to be calculated.
- The containers are stored and ordered evenly distributed as given by a European material handling provider.
- The container to be relocated is relocated to the nearest available storage location.
- The filling degree is limited to a specific value to ensure that relocations can always be done.

The notation used in this approach can be found in Table 1.

**Table 1:** Notation of the analytical approach.

$\Delta x$	Distance between two grid elements along the x-axis
$\Delta z$	Distance between two grid elements along the z-axis
*a*	Acceleration/deceleration rate of a robot
*E*(*CT*)	Expectation of one robot’s cycle time
*f*	Filling degree
$h_{C}$	Height of a storage container
$k_0$	Position of the picking station along the x-axis
$n_x$	Number of grid elements along the x-axis
$n_z$	Number of grid elements along the z-axis
*sh*	Storage height of a container stack
$t_{CX}$	Time for the container exchange at the picking station
$t_{IO\_DCC}$	Time required at the I/O shaft in a dual command cycle
$t_{IO\_SCC}$	Time required at the I/O shaft in a single command cycle
$t_{L}$	Time to lock and unlock the container before/after picking up/dropping off a container
$t_{R\_rel}$	Time of a robot required to travel at the relocation cycle
$t_{R\_DCC}$	Additional time of a robot to travel in a dual command cycle
$t_{R\_SCC}$	Time of a robot required to travel in a single command cycle
$t_{T}$	Time required to transfer a container up from or down onto the stack by lifting or lowering
$t_{WX}$	Time of a robot to change the wheels from one direction to another
$v_{R}$	Velocity rate of a robot in horizontal direction
$v_{T}$	Velocity rate of a robot for lifting and lowering
$w_{rel}$	Probability of a relocation cycle

**Figure 4:**
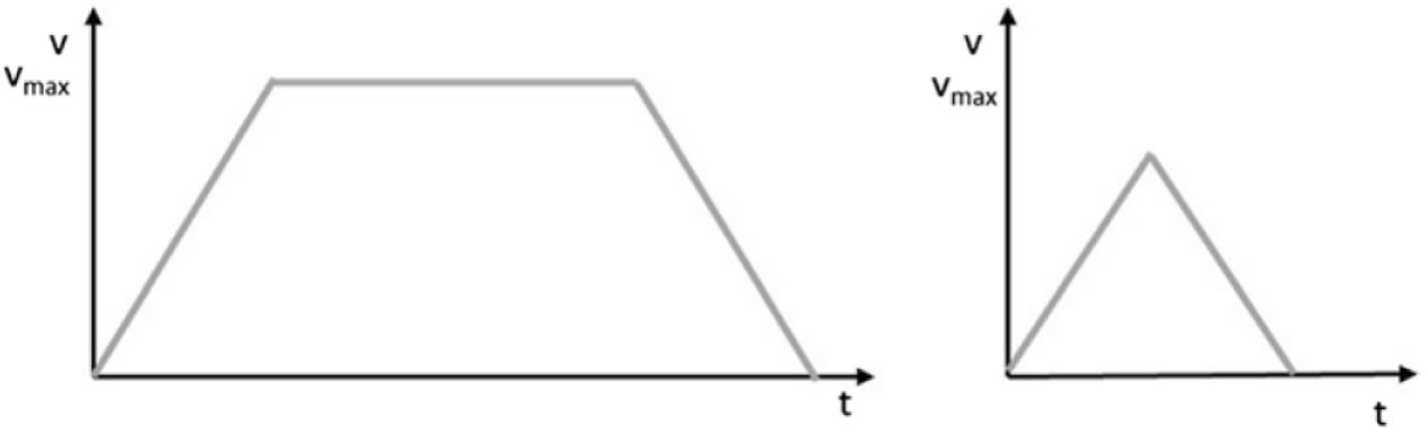
Trapezoid or triangular drive^34^.

Since this approach considers robots with non-constant velocity rates, a distinction for the time function must be made. Depending on the travel distance, either the maximum speed is reached, and thus the trapezoidal drive mode takes place as it can be seen in Fig. 4 on the left side or not in the case of short distances, the triangular drive (right graph). The following approach requires the time function for every mean ride time.

As long as the maximum velocity is not reached, hence $l<\frac{v_{R}^{2}}{a}$ (right graph in Fig. 4), the time function arises to:

1 $$\begin{aligned} {\textbf{t}}(l)=2\sqrt{\frac{l}{a}} \end{aligned}$$

Therein, ${\textbf{t}}$ is a function of the variable *l* describing the robot’s ride distance. At the moment the robot is accelerated to its maximum velocity, the time function for the trapezoidal drive mode is then calculated by:

2 $$\begin{aligned} {\textbf{t}}(l)=\frac{l}{v_{R}}+\frac{v_{R}}{a} \end{aligned}$$

### Cycle time calculations

**Figure 5:**
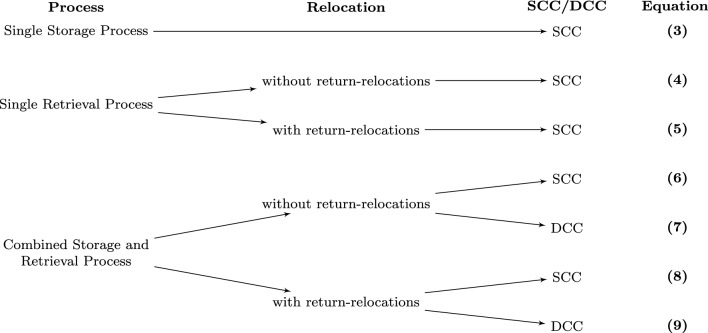
Overview of the cycle time calculation options.

RCS/R systems usually operate in a dual command cycle as described in section "RCS/R system description". Though, other operation modes are conceivable and necessary. The time calculation depends on whether storage or retrieval occurs, the operating mode (SCC or DCC), and strategies used within the warehouse. Figure 5 gives an overview of the different processes and which of the following seven equations has to be used:

The expectation of one robot’s cycle time *E*(*CT*) calculates, depending on the corresponding operation mode, to:

1. Expectation of the cycle time for storage process performing in a single command cycle.
  3 $$\begin{aligned} E(CT)_{SCC\_Stor}=2\cdot t_{R\_SCC}+t_{T}+t_{IO\_SCC} \end{aligned}$$
2. Expectation of the cycle time for retrieval process without return relocations performing in a single command cycle.
  4 $$\begin{aligned} E(CT)_{SCC\_Retr}=2\cdot t_{R\_SCC}+t_{T}+w_{rel}\cdot (t_{R\_rel}+2\cdot t_{T})+t_{IO\_SCC} \end{aligned}$$
3. Cycle time calculation for retrieval process with return relocations performing in a single command cycle.
  5 $$\begin{aligned} E(CT)_{SCC\_Retr\_RR}=4\cdot t_{R\_SCC}+t_{T}+2\cdot w_{rel} \cdot (t_{R\_rel}+2\cdot t_{T})+t_{IO\_SCC} \end{aligned}$$
4. Expectation of the cycle time for combined storage and retrieval process without return relocations performing in a single command cycle.
  6 $$\begin{aligned} E(CT)_{SCC\_Stor+Retr}=4\cdot t_{R\_SCC}+ 2 \cdot t_{T}+w_{rel}\cdot (t_{R\_rel}+2\cdot t_{T})+2\cdot t_{IO\_SCC} \end{aligned}$$
5. Expectation of the cycle time for combined storage and retrieval process without return relocations performing in a dual command cycle.
  7 $$\begin{aligned} E(CT)_{DCC\_Stor+Retr}=2\cdot t_{R\_SCC}+t_{R\_DCC}+2\cdot t_{T}+ w_{rel}\cdot (t_{R\_rel}+2\cdot t_{T})+t_{IO\_DCC} \end{aligned}$$
6. Expectation of the cycle time for combined storage and retrieval process with return relocations performing in a single command cycle.
  8 $$\begin{aligned} E(CT)_{SCC\_Stor+Retr\_RR}=6\cdot t_{R\_SCC}+2\cdot t_{T}+2\cdot w_{rel}\cdot (t_{R\_rel}+2\cdot t_{T})+2\cdot t_{IO\_SCC} \end{aligned}$$
7. Expectation of the cycle time for combined storage and retrieval process with return relocations performing in a dual command cycle.
  9 $$\begin{aligned} E(CT)_{DCC\_Stor+Retr\_RR}=2\cdot (t_{R\_SCC}+t_{R\_DCC}+t_{T}+ w_{rel}\cdot (t_{R\_rel}+2\cdot t_{T}))+t_{IO\_DCC} \end{aligned}$$

The individual time components result from the following sections discussing the mean ride times $t_R$ (section "Mean ride time"), the transfer times $t_T$ (section "Mean transfer time for container lifting and lowering"), the picking times at the I/O shaft $t_{IO}$ (section "Mean time at the I/O shaft"), and the relocation probability $w_{rel}$ (section "Relocation cycle").

### Mean ride time

The basic robot ride in an SCC is from/to the I/O shaft to/from a storage stack along the grid. Therefore, the travel time can be calculated with equation 10. Depending on the distance to ride, either equation 1 or 2 has to be used for the correct time function. Equation 10 is based on the first CTM for AS/RS, was expanded SBS/RS by Eder^17^, and is now adapted for RCS/RS. Especially the second sum to consider the two-directional movement of the robot along the grid and the ${{\,\text{sign}\,}}$ function for the possible wheel exchange $t_{WX}$ were added.

10 $$\begin{aligned} t_{R_\_SCC}=\frac{1}{n_x}\cdot \frac{1}{n_z}\cdot \sum \limits _{k=1}^{n_x}\sum \limits _{l=1}^{n_z} {\textbf{t}}(l\cdot \Delta z)+ {\textbf{t}}((\vert k-k_0 \vert ) \cdot \Delta x)+ t_{WX} \cdot {{\,\text{sign}\,}}(\vert k-k_0 \vert ) \end{aligned}$$

The first term after the double sum describes the ride along the z-direction. Without a directional change and thus without a wheel change ($\vert k - k_0 \vert = 0$), the other two terms result in zero. Therein, $k_0$ describes the position of the I/O shaft along the x-axis, and the variables *k* and *l* are the summation indices. If the direction has to be changed once, i.e. the robot also drives along the x-axis, this must be considered with an additional time component. So, for $\vert k - k_0 \vert > 0$, the last term calculates to ${{\,\text{sign}\,}}(\vert k-k_0 \vert )=1$ since the ${{\,\text{sign}\,}}$ function results in one for every value greater than zero.

In the case of a DCC, the robot has a connection ride between the storage and the retrieval stack. Therefore, analogous to Eq. 10 for an SCC, the following expression (Eq. 11) represents the travel time from the storage stack to the retrieval stack.

11 $$\begin{aligned} t_{R_\_DCC}=\frac{1}{n_x^2}\cdot \frac{1}{n_z^2}\cdot \sum \limits _{k=1}^{n_x} \sum \limits _{l=1}^{n_x} \sum \limits _{m=1}^{n_z} \sum \limits _{n=1}^{n_z} {\textbf{t}}((\vert m-n \vert ) \cdot \Delta z ) + {\textbf{t}}((\vert k-l \vert ) \cdot \Delta x ) +t_{WX}\cdot {{\,\text{sign}\,}}((\vert k-l \vert ) \cdot (\vert m-n \vert )) \end{aligned}$$

Again, the first term after the quadruple sum describes the ride along the z-, and the second term stands for the ride along the x-direction. A wheel exchange is indispensable if neither the first nor the second term calculates to zero, i.e. for $\vert k-l \vert \ne 0$ or $\vert m-n \vert \ne 0$.

### Mean transfer time for container lifting and lowering

When a robot arrives at an assigned stack to store or retrieve a container, the time to lift or lower the container depends on the actual stack height of the corresponding stack, i.e. on the filling degree of the storage system, on the velocity of the lifting and lowering device, and on the maximum stack height. Eder^17^ developed a closed expression for storage depths up to five containers and any filling degree. His formula was adapted and slightly modified for RCS/RS. It can now be applied for a theoretically unlimited number of stack containers to calculate the mean transfer time for the lifting and lowering the containers.

12 $$\begin{aligned} t_{T}=t_{L}+ \sum \limits _{n=1}^{sh} \sum \limits _{i=0}^{n-1} \frac{1}{sh+4\cdot i}\cdot \begin{pmatrix}sh-1\\ i\end{pmatrix} \cdot f^{sh-1-i}\cdot (1-f)^i \cdot 2 \cdot \frac{h_{C}}{v_{T}} \cdot n \end{aligned}$$

Therein, $t_{L}$ is the time required to lock or unlock the locking claws of the lifting device. *f* is the filling degree of the storage system. On the one hand, the first term after the binomial coefficient describes the probability of a full stack. On the other hand, the second term characterises the probability of an empty stack. A precise and more detailed description of all terms can be found in Eder^17^. This equation is used both for storage and retrieval and for the transfer in case of relocations.

### Relocation cycle

If the stack height is $sh>1$, it is possible that relocations must be done to be able to retrieve the required container.

#### Relocation probability

The probability of a relocation depends on the space available and the maximum stack height. The following equation is based on Eder’s approach^33^ for SBS/RS:

13 $$\begin{aligned} w_{rel}=\sum \limits _{n=0}^{sh-2} \sum \limits _{i=1}^{sh-1-n}\cdot \frac{i}{sh-n} \cdot \begin{pmatrix} sh\\ n \end{pmatrix} \cdot f^{sh-n}\cdot (1-f)^n \end{aligned}$$

Inserting $sh=2$, the probability of a possible relocation simplifies to $w_{rel}=\frac{1}{2}\cdot f^2$. Eder^33^ describes the factors as the following: $f^2$ represents the probability that both stacks are occupied by totes. The necessity for relocation comes from the factor $\frac{1}{2}$ because the first stored tote has to be retrieved.

#### Relocation ride time

First, a relocation strategy has to be chosen to calculate one robot’s ride time for a relocation. This paper assumes a ’one path’ relocation strategy to minimise the number of directional changes and, thus, the necessary wheel exchanges. Since RCS/RS are typically built quadratically and the number of stacks along the two horizontal axes is much higher than the number of edges and corners, the assumption of four possible paths leading away from the order stack can be made.

**Figure 6:**
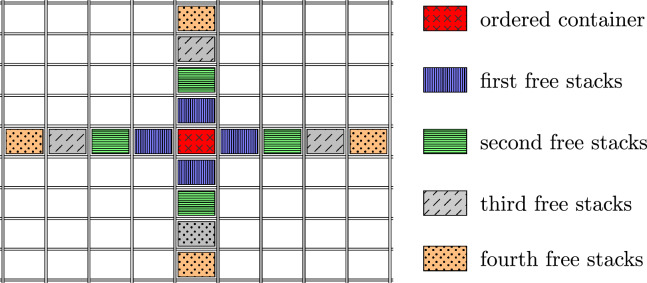
Relocation distance and next free stacks in the middle of the grid.

Figure 6 depicts the relocation situation for a relocation cycle anywhere on the grid.

As shown in Fig. 6, the robot is supposed to transport the relocation container to one of the stacks along one of the four axes. Starting with the first four stacks (blue) directly neighboured. If all of them are occupied, the following four stacks (green) are checked for free space. This process is repeated until a stack with space for another container is found.

This procedure can be explained due to the acceleration/deceleration rate and the time required for the wheel exchange. It can be advantageous to only ride along one axis without directional changes. In the case of a robot with a velocity rate of $v_R=3 \frac{m}{s}$ and an acceleration/deceleration rate of $a=0.8 \frac{m}{s}$, it is shorter to ride to the seventh stack along one direction instead of relocating to the directly neighbouring stack along the diagonal which requires a wheel exchange.

Based on the probability of a relocation, the robot’s time for a relocation ride can be calculated. That, again, depends on the number of accessible storage locations next to the stack where the retrieval has to be done. Equation 14 depicts the relocation ride time.

14 $$\begin{aligned} t_{R\_rel}= & {} \sum \limits _{i=1}^{n_x} \sum \limits _{j=1}^{n_z} \frac{(f^{sh})^{X}\cdot (1-(f^{sh})^{Y})}{{{\,\text{max}\,}}(i,j)} \cdot 2 \cdot {{\,\text{max}\,}}({\textbf{t}}((i) \cdot \Delta x); {\textbf{t}}((j) \cdot \Delta z)) \end{aligned}$$

With:

15 $$\begin{aligned} X = 2\cdot ({{\,\text{max}\,}}(i,j)-1)\cdot {{\,\text{max}\,}}(i,j) \end{aligned}$$

And:

16 $$\begin{aligned} Y = 4\cdot {{\,\text{max}\,}}(i,j) \end{aligned}$$

The first term $(f^{sh})^{X}$ describes the probability that the four directly neighbouring stacks are fully occupied. Therein, $f^{sh}$ represents the probability that one stack is full up its maximum height, and the exponent describes the total number of occupied stacks. Analogous, the second expression $(1-(f^{sh})^{Y})$ returns the probability of a free storage location on a stack within those four stacks. The exponent represents the number of storage slots within the same relocation area. On the one hand, the term ${{\,\text{max}\,}}(i,j)$ in the denominator guarantees that the denominator will not get zero. On the other hand, it ensures that all stacks where the relocation containers could be relocated are considered.

Referring to Fig. 6, the stacks to where the relocation containers are transported are arranged along the four paths away from the retrieval stack. The number of stacks within the first relocation area ($Y=4\cdot {{\,\text{max}\,}}(i,j)$) describes all the four blue stacks (first free stacks). The number of occupied stacks results by using the Gaussian sum formula $\frac{n \cdot (n-1)}{2}$ as can be seen in the exponent *X*.

The worst-case scenario is the assumption of low stack heights (e.g. $sh=2$) and very high filling degrees (e.g. $f=0.99$). Almost this very unrealistic case leads to a free stack within each of the five neighbouring grid elements with a probability of more than 91%. Riding nine relocation stacks along one of the four directions, there is a free stack with a probability of 99.9%. Real RCS/RS usually have stack heights from $sh=10$ up to $sh=25$ or even higher and filling degrees from 75% to 90%, thus a very high probability of finding a free storage location within the first neighbouring stacks.

### Mean time at the I/O shaft

RCS/R systems usually operate in a DCC as described in section "RCS/R system description". Though, other operation modes are conceivable and necessary. In an SCC, the robot lowers the container, opens the locking claw and lifts the empty device. Equation 17 describes the robot’s time required on the I/O shaft in an SCC:

17 $$\begin{aligned} t_{IO\_SCC}=t_{L} + 2\cdot \frac{h_{C}}{v_{T}} \cdot sh \end{aligned}$$

The fraction depicts the time for lifting and lowering the container through the shaft depending on the container height, the velocity of the lifting device, and the stack height. It is considered twice because the mechanism must be lifted after dropping the container off.

Operating in a dual command cycle, the robot is supposed to wait for another container to store after retrieving a container. This is taken into account in equation 18 by $t_{CX}$, which stands for the container exchange time in the picking station, as well as by multiplying $t_{L}$ by two due to the unlocking of the retrieval container and the locking of the storage container.

18 $$\begin{aligned} t_{IO\_DCC}=2 \cdot t_{L} + t_{CX}+ 2\cdot \frac{h_{C}}{v_{T}} \cdot sh \end{aligned}$$

The container exchange in the picking station can be done, e.g. by a belt conveyor or a rotary table. The time for locking the claws has to be multiplied by two since after opening the claws another container has to be picked up.

## Numerical study

The fifth section is supposed to validate the analytical approach from section "Analytical approach" with a discrete event simulation of an RCS/RS, which will be done in section "Validation and verification" and to test different parameters and configurations (section "Parameter variations"). To close the numerical study, an optimisation example shall be done (section "Optimisation example") in order to show how the analytical approach can be implemented and used. Table 2 presents the input parameters for the numerical study.

**Table 2:** Parameters for the RCS/RS.

Parameter	Value
Number of grid elements along the x-axis	$n_x \in \{10,20,25,30,40,50\}$
Number of grid elements along the z-axis	$n_z \in \{5,10,20,25,30,40,50\}$
Storage height of a container stack	$sh \in \{1... 25...(100)\}$
Filling degree	$f\in \{10\%,25\%,50\%,75\%,90\%,95\%,98\%\}$
Container height	$h_{C}=330$ mm
Robot horizontal velocity rate	$v_{R}=3 \frac{{\text{m}}}{{\text{s}}}$
Robot lifting and lowering velocity rate	$v_{T}=1.6\frac{{\text{m}}}{{\text{s}}}$
Robot horizontal acceleration / deceleration rate	$a=0.8\frac{{\text{m}}}{{\text{s}}^2}$
Container exchange time at the picking station	$t_{CX}=3$ s
Robot time to lock/unlock the container	$t_{L}=1$ s
Robot wheel change time	$t_{WX}=1$ s

The main focus will be the throughput of one robot within the considered system. Regarding Fig. 5 from section "Cycle time calculations", the system investigated is operating in a dual command cycle, and the cycle time is calculated with equation 7, which represents one of the classic operating modes of an RCS/RS. Whether return relocations should be done or not can be answered by the fact that those would just mean an additional time for the robot’s cycle time since this paper considers an inhomogeneous article distribution with a homogeneous, equally distributed access structure over the stack height.

For the validation of the analytical approach of section "Analytical approach", the results will be compared with those of 20 independent scenarios of the simulation model. The simulation model—rebuilding an RCS/RS with the processes controlling the system in the background—was created in the DES simulation software SIMIO (version 14). The containers were evenly distributed over all stacks and storage heights. The process within the simulation can be summarised by the three main processes within a multiple-deep storage system: Storage, Relocation, and Retrieval. Based on that, a new unit to be stored arrives at the picking station, which represents the I/O point of the simulation system and gets assigned to the robot after capturing the according data. As soon as the robot is available, the new tote gets lifted through the I/O shaft up to the grid level and transported to the, by the control logic before assigned—randomly chosen—stack. After dropping off the container at the correct stack, the storage process is finished. The robot is now allocatable for the next task. In case of an order without direct access, because other containers are stacked above the required one, all of them have to be relocated. The simulation logic, therefore, uses the ’one path’ relocation strategy, assigning the following free stack along one of the four outgoing paths. After relocating all of the blocking containers, the required one can be lifted, transported to, and lowered down through the I/O shaft by the robot. When picking up a new storage container, the retrieval process is done.

All the parameters from Table 2, such as the number of stacks along both horizontal axes, the stack height, the filling degree, etc., can be varied in the simulation.

### Validation and verification

This section shall validate the analytical approximation from section "Analytical approach". The complexity of the system will be limited in the beginning to be sure the following check steps can be confirmed:

- Robot ride time
- Probability for relocation
- Relocation ride and lifting and lowering time

First, the stack height will be set to $sh=1$ for several quadratic and rectangular grid sizes to check the robot’s ride time on the grid. Afterwards, the relocation probability shall be checked. This will be done by analysing the results from Eq. 13 with the mean number of relocations necessary per retrieval recorded in the DES. The stack height will be varied up to 25; to theoretically validate the formula, additional also up to 100. To prove the correctness of the analytical equation regarding the lifting and lowering as well as the relocation ride time, the throughput depending on the stack height will be under investigation for different grid sizes.

The results from the analytical approach are compared with those from the DES to validate the developed approximations. Moreover, the relative approximation error is calculated by equation 19:

19 $$\begin{aligned} \varepsilon = \frac{\vert \nu _A - \nu _{DES} \vert }{\vert \nu _{DES} \vert } \quad \text{ for } \quad \nu _{DES} \ne 0 \end{aligned}$$

**Figure 7:**
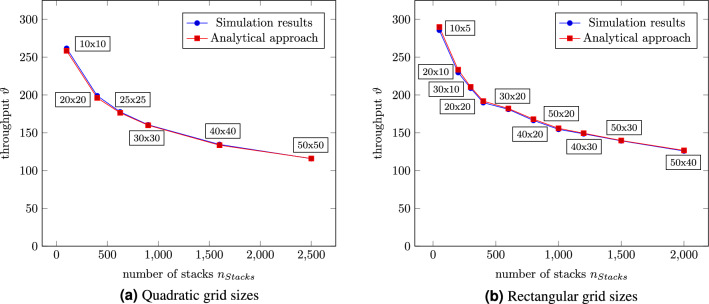
Throughput of an RCS/RS for a stack height of $sh=1$ for different grid sizes and a filling degree of 90%.

Firstly, the time required for a ride will be under investigation. Thus, the stack height is limited to one. Figure 7a exposes one robot’s throughput for different quadratic grid sizes and a stacking height limited to one, i.e. no relocations. The numerical results regarding the throughput are compared to the analytical, and, as shown in Fig. 7a, the discrepancy converges towards zero. Similar results for rectangular grids can be seen in Fig. 7b. The throughput decreases for larger grid sizes, regardless of whether quadratic or rectangular arrangements. The results both from the analytical approach and the DES and the estimation error can also be found in Table 3.

As shown in Fig. 7, the possible throughput decreases with increasing stacks. Comparing the quadratic grids (Fig. 7a) with the rectangular (Fig. 7b), it can observed that a quadratic storage system enables higher throughputs than a rectangular for the same number of stacks. E.g. a 20 by 20 grid leads to a nearly 4% higher performance than the 40 by 10 system.

**Table 3:** Comparison of the results from the analytical approach vs. discrete event simulation and the relative estimation error.

Grid size	Analytical approach	Discrete event simulation	Estimation error	Grid size	Analytical approach	Discrete event simulation	Estimation error
	$\vartheta [{1}/{h}]$				$\vartheta [{1}/{h}]$		
10x5	289.9	285.6	1.5%	40x10	191.9	189.8	1.1%
10x10	261.7	258.3	1.3%	40x20	168.0	166.3	1.0%
20x10	233.3	229.6	1.6%	40x30	149.4	148.6	0.5%
20x20	198.9	196.1	1.4%	40x40	134.5	133.5	0.7%
25x25	177.6	176.4	0.7%	50x20	155.8	154.5	0.9%
30x30	160.5	159.9	0.4%	50x30	139.7	139.3	0.3%
30x10	210.6	208.9	0.8%	50x40	126.6	125.9	0.6%
30x20	182.1	181.0	0.6%	50x50	115.8	115.9	0.1%

**Figure 8:**
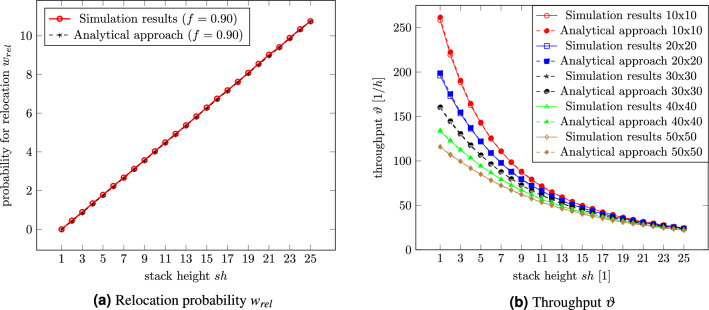
Number of necessary relocations for one retrieval depending on the stack height for a filling degree of 90% and the corresponding throughput for different grid sizes.

Based on the ride time for a single deep RCS/RS, the next step will be having multiple containers stacked on each other. Eders^17^ formula determining the probability of relocations was expanded to, for RCS/RS typical, storage heights and plotted over the stack height for a filling degree of 90% in Fig. 8a. Figure 8b depicts the corresponding throughput depending on the stack height for different grid sizes and compares the results from the analytical approach with those from DES. According to Eq. 13, the relocation probability from the left figure is independent of the grid size.

The left Fig. 8a shows a linear increasing relocation probability for an ascending stack height. While the grid size does not impact the relocation probability, a lower filling degree would reduce the probability since the stacks are not that full compared to fuller warehouses. The biggest relative estimation error is about 5% at a stack height of $sh=2$. This can be explained by the total number of retrievals of the simulation. The DES stops at 10,000 retrievals, which means only about 4000 relocations compared to more than 100,000 relocations at a stack height of $sh=25$.

Figure 8b depicts the throughput depending on the stack height for different quadratic grid sizes from 100 to 2500 stacks. The smaller the grid, the steeper the curves fall. All curves converge beginning at a stack height of about $sh=14$. Regardless of the grid size, one robot can retrieve about 23 containers per hour at a stack height of $sh=25$. Reducing the stack height to $sh=14$ doubles the throughput.

**Figure 9:**
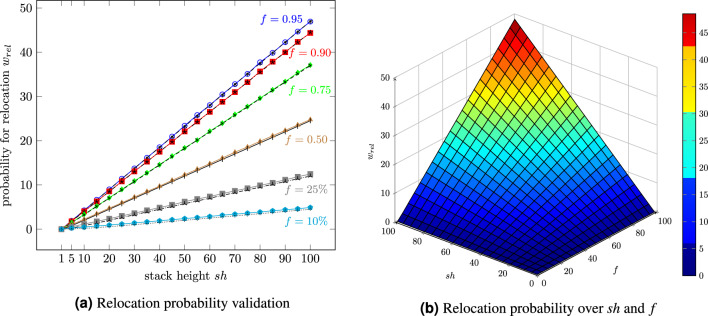
Number of necessary relocations for one retrieval up to a theoretical stack height of 100.

At this point, the question arises whether purely fictitious, even larger stack heights are possible. For this reason, the relocation probability will be tested for storage heights up to a theoretical stack height of $sh=100$. A grid size of 50 by 20 with 1000 stacks guarantees that every relocation can get a relocation storage location. Figure 9a confirms the assumption that equation 13 has validity up to large stack heights. The for RCS/RS untypical low filling degrees and low stack heights have the largest estimation errors. Again, this can be explained by the total number of retrievals of the simulation. Claiming, e.g., 100,000 retrievals instead of 10,000 would reduce the error, but the computation time would be too long.

Although the DES took long to compute and simulate such a big storage system (maximum 95,000 containers), it was possible to gain statements and to validate the analytical formula (equation 13). The estimation error is tiny, and the biggest error occurs at a filling degree of 10%. Figure 9a shows that the relocation probability $w_{rel}$ is linear over the stack height independent of the filling degree.

The right plot (Fig. 9b depicts the relocation probability displayed over the stack height and the filling degree.

### Parameter variations

**Figure 10:**
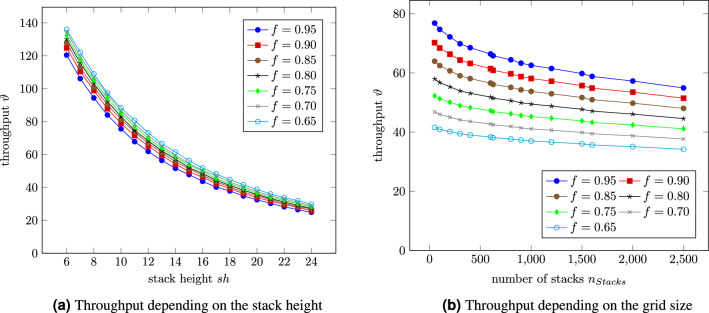
Throughput of a 10 by 10 RCS/RS for different filling degrees in dependence of the stack height and the grid size.

Since RCS/RS usually operate in a dual command cycle with stack heights from $sh=6$ to $sh=24$ and high filling degrees up to a maximum of 95%, further study will now provide some evaluations focusing on those stack heights and filling degrees to gain insights on how those systems can be deployed. Figure 10a presents the throughput of a 10 by 10 RCS/RS depending on the stack height for different filling degrees. All results within this subsection are gained out of the analytical approach. Figure 10b keeps the stack height constant while varying the grid size and shows the throughput depending on the grid size with a constant maximum stack height of $sh=16$ for different filling degrees:

The number of picks per hour depends on the stack height and is coloured plotted for different filling degrees within the storage system. As can be seen, the throughput decreases for higher stack heights. The highest filling degrees also have the largest decreasing rate of throughput, i.e. the steepest curves. The number of relocations can explain this effect. The curves describing the relocation probability for lower filling degrees are flatter compared to the ones for higher filling degrees (Figs. 8a and 9a).

While Fig. 10a shows the impact of the stack height on the system’s performance, Fig. 10b focuses on the grid size and thus on the number of stacks, which is plotted along the x-axis. The smallest system under investigation is once more a 10 by 5 grid, i.e. 50 stacks. The number of stacks was successively increased up to 2500, i.e. a 50 by 50 grid with a maximum of 40,000 containers to be stored. High filling degrees result in smaller throughputs nearly independent of the grid size, as can be seen by the bottom curve (95% filling degree). Figure 10b shows that the influence of the grid size is reduced more and more the higher the filling degree gets. An explanation, therefore, is the broader grid and, thus, the longer ride times from and to the I/O shaft, which also decreases the impact of the high temporal portion of the relocations. In contrast, a low number of stacks combined with a small filling degree leads to a high throughput.

**Figure 11:**
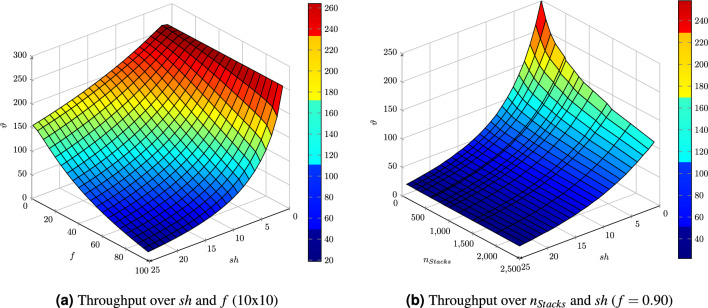
Throughput of an RCS/RS with varying stack height, grid size, and filling degree.

Since there are hardly any quantitative statements on the system performance of RCS/RS, the last part of this subsection shall provide some parameter variations to discuss the system’s characteristics. Therefore, the system’s throughput will be plotted over the stack height and the filling degree. Figure 11a depicts the throughput of a 10 by 10 RCS/RS with the parameters according to Table 2. Moreover, the impact of the grid size on the throughput shall be displayed, which is depicted with Fig. 11b showing the throughput over the number of stacks and the stack height for a filling degree of 90%.

As expected, the throughput decreases for increasing stack heights and filling degrees. The robot never reaches its maximum velocity within a 10 by 10 RCS/RS. Thus, the ride’s impact is not as high as for larger grids. High filling degrees and high stack heights enable high storage densities but also lead to low throughput rates, as can be seen in the left Fig. 11a. Figure 11b on the right side shows that the performance decrease is nearly linear and independent of the grid size starting at a stack height of $sh=8$.

### Optimisation example

The third part of the numerical study is aimed to show the usage of the analytical approach with a little optimisation example. The basic requirement is a storage capacity for about $N=5000$ containers. Table 4 presents the required input data:

**Table 4:** Input data for the optimisation example.

Parameter	Value
Number of stacks	$n_{Stacks}=\in \{4,750,...,5,250\}$
Storage height of a container stack	$sh \in \{8,9,...,25\}$
Filling degree of the storage system	$f\in \{85\%,...,90\%,...,95\%\}$
Throughput required	$\vartheta _{min}=70 \ {1}/{h}$

A storage system with a capacity of $N=5000$ containers with a maximum deviation of +/- 5% shall be designed optimally. The stack height can, therefore, be in the—for RCS/RS typical—range from $sh=8$ to $sh=25$; the filling degree shall ideally be not smaller than 85% and not larger than 95%. A minimum of 70 containers has to be retrieved per hour. The optimisation example is now supposed to give answers to the following questions:

- Is there a modification that enables such a performance?
- If yes, how does the system configuration look like?
- If no, how big is the maximum throughput for the required storage capacity?

The results deployed from the analytical approach are summarised in Table 5 sorted by the number of stacks.

**Table 5:** Optimisation example for RCS/RS with one robot.

	Storage system			Capacity *N*			Throughput $\vartheta [{1}/{h}]$			Area
	Grid	$n_{Stacks}$	*sh*	88%	90%	93%	88%	90%	93%	$A [\text{m}^2]$
1	30x10	300	18	4752	4860	5022	39.8	38.0	35.4	105
2	20x20	400	14	4928	5040	5208	53.0	51.0	48.1	140
3	30x20	600	9	4752	4860	5022	79.1	77.0	73.8	210

**Figure 12:**
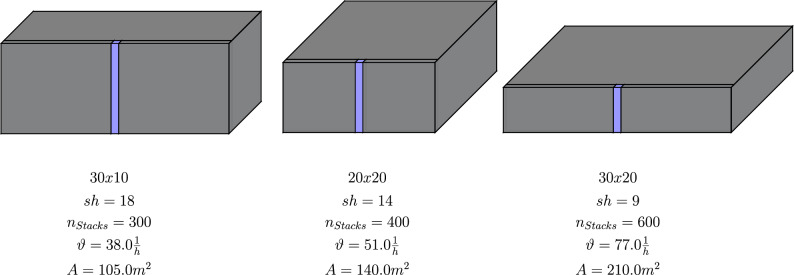
Optimisation example.

As can be seen in Table 5, three results could be gained for this scenario. All three results are also graphically depicted in Fig. 12.

The first one has the most minor space demand ($A=105\,\text{m}^2$) since it has container stacks up to $sh=18$ but also the smallest throughput with only about 35 to 40 retrievals per hour. The third variant can achieve the best output performance with about 74 to 80 containers per hour, representing a 30 by 20 grid with a stack height of $sh=9$. This also goes ahead with a twice as high space demand as the first mentioned system. A good compromise between space demand and performance could be the version with a 20 by 20 grid with a stack height of $sh=14$, enabling throughputs of about 50 order picks per hour and a space demand of $A=140\,\text{m}^2$.

To answer the above-listed questions: Yes, a modification enables a throughput of more than 70 retrievals per hour. The needed configuration would be a 30 by 20 RCS/RS with 9 containers stacked above each other, resulting in a dependence on the filling degree, storage capacity of 4750 to 5020 and a space demand of 210 m^2^.

## Conclusion and outlook

RCS/RS have many advantages besides the high storage density, which is obviously one of the reasons for sales. Due to the absence of real competition in the market in previous times, there are hardly any statements on the performance of such systems. Moreover, not just the providers keep the data secrets. Just a few scientific papers exist that deal with this topic. Most had specific targets for default system settings. The only relevant paper presented an analytical approach using a SOQN, which is neither easy nor fast solvable analytically. Indeed, some other storage systems have similar characteristics regarding the storage and retrieval process, movement of the totes along the three axes or the system’s logic. SBS/RS and RCS/RS especially have a close connection since such systems can also have vehicles serving multiple tiers of a multiple-deep storage rack. Nonetheless, there are some differences.

This paper aimed to present a fairly accurate analytical approach to calculate the performance of RCS/RS using one robot on the grid. This was done using a CTM based on the multiple-deep SBS/RS performance calculation to predict the system’s throughput. Many system parameters, such as the spatial size, how many containers get stacked on each other, and the type of the picking station, greatly influence the cycle time or the container type. Still, the filling degree of the storage system or the technical data for the robot can also be chosen. The probability-based relocation approximation was adjusted and then validated up to purely fictitious storage heights of one hundred. This is a novelty since the relocation probability and the travel time for several similar storage systems with multiple deep stacks or racks can now be calculated easily.

The accuracy of the analytical model was validated by comparing the results with those from a DES. Based on the validation, a parameter variation was done. It could be seen that high filling degrees and high stack heights go ahead with small throughputs. The grid size does not significantly impact, especially for high filling degrees and, above all, for high storage heights. Additionally, an optimisation example was examined to show the formula tool’s practical relevance, providing results quickly and straightforwardly.

The great parameter variability enables the prediction of the performance of several different RCS/R systems, including other design configurations. Another big advantage is the easy applicability to business and industry close software such as standard table calculation or computer algebra tools. Customers can now estimate one robot’s expected throughput and check the data offered by the material handling providers. Apart from that, the approach helps the providers save time and computational power exhausted by intense simulations as done nowadays to predict the possible throughput. Furthermore, from a scientific point of view, this approach to calculating the performance of one robot within an RCS/RS can be used as a first step in designing such a system. The approach is valid for a theoretically unlimited stack height. It provides easy-to-solve cycle time calculation formulas, which constitute the basis for expanding the system with multiple operating robots.

The next step is adding more robots and picking stations to the analytical approach to give an outlook on further work. The questions of when an obstacle of two or more robots happens or when a queue at the I/O shaft forms will be mandatory. Another topic of interest could be considering an article distribution (e.g. class-based article distributions) to improve the system performance due to fewer relocations. Based on the assumptions made in section "Analytical approach" regarding the assigned relocation stacks, a time-window-based approach for always selecting the best stack to relocate to could give a more precise statement about the relocation cycle. Moreover, the resulting costs for such a storage system could be interesting. All those targets will be part of future scientific discourses, which shall support the design process of RCS/RS.

### Supplementary Information

Supplementary Information.

## Data Availability

The datasets generated during and/or analysed during the current study are available from the corresponding author upon reasonable request.
