# Supplementary material for: An analytical performance approach for RCS/RS with one robot serving multiple stack heights under a one-path relocation strategy
Source: Sci Rep. 2024 Feb 13;14:3593. doi: 10.1038/s41598-024-53884-6 (PMC10864412; doi:10.1038/s41598-024-53884-6)
Supplement: Supplementary file 1 — Supplementary Information. [file 41598_2024_53884_MOESM1_ESM.pdf]

Appendix

Table 6. Comparison of the results from the analytical approach vs. discrete event simulation and the relative estimation error.

		Analytical approach						Discrete event Simulation						Estimation error					
		10x10	20x20	30x30	40x40	50x50	*w_rel*	10x10	20x20	30x30	40x40	50x50	*w_rel*	10x10	20x20	30x30	40x40	50x50	*w_rel*
sh	1	261.7	198.9	160.5	134.5	115.8	0.00	258.3	196.1	159.9	133.5	115.9	0.00	1.3%	1.4%	0.4%	0.7%	0.1%	0.0%
	2	222.6	175.5	144.9	123.3	107.4	0.41	219.3	173.1	143.6	122.7	106.6	0.45	1.5%	1.4%	0.9%	0.6%	0.7%	5.8%
	3	190.5	154.9	130.6	112.8	99.3	0.85	188.6	153.5	129.8	112.3	99.6	0.89	1.0%	1.0%	0.6%	0.4%	0.2%	4.0%
	4	164.6	137.3	117.9	103.2	91.8	1.30	162.7	136.3	117.0	103.5	91.7	1.34	1.2%	0.7%	0.7%	0.2%	0.1%	3.3%
	5	143.3	122.2	106.5	94.4	84.8	1.75	142.7	121.9	106.7	94.3	85.0	1.78	0.5%	0.3%	0.2%	0.2%	0.2%	1.7%
	6	125.8	109.2	96.5	86.5	78.3	2.20	124.9	108.9	96.6	86.9	78.1	2.25	0.6%	0.3%	0.1%	0.5%	0.3%	2.1%
	7	111.1	98.0	87.6	79.3	72.4	2.65	110.7	97.7	87.5	79.2	72.5	2.69	0.4%	0.2%	0.1%	0.1%	0.2%	1.6%
	8	98.8	88.3	79.8	72.8	66.9	3.10	98.5	87.7	80.1	72.7	67.2	3.10	0.3%	0.7%	0.4%	0.1%	0.4%	0.1%
	9	88.3	79.8	72.8	67.0	62.0	3.55	87.5	79.3	72.6	67.0	62.4	3.59	1.0%	0.7%	0.3%	0.1%	0.6%	1.2%
	10	79.4	72.5	66.7	61.7	57.4	4.00	78.9	72.1	66.7	62.1	57.6	4.06	0.7%	0.6%	0.0%	0.7%	0.3%	1.4%
	11	71.8	66.1	61.2	57.0	53.3	4.45	71.4	66.2	61.4	56.6	53.4	4.49	0.6%	0.2%	0.4%	0.7%	0.2%	0.9%
	12	65.2	60.4	56.3	52.7	49.6	4.90	64.7	60.3	56.6	53.0	50.1	4.91	0.7%	0.2%	0.5%	0.4%	1.1%	0.1%
	13	59.4	55.4	52.0	48.9	46.2	5.35	59.2	55.1	52.1	48.9	46.2	5.37	0.4%	0.7%	0.2%	0.1%	0.1%	0.3%
	14	54.4	51.0	48.1	45.4	43.1	5.80	54.0	50.7	48.6	45.3	43.6	5.84	0.6%	0.7%	1.0%	0.4%	1.1%	0.7%
	15	49.9	47.1	44.6	42.3	40.3	6.25	49.6	47.2	44.6	42.3	40.2	6.28	0.8%	0.1%	0.0%	0.0%	0.2%	0.4%
	16	46.0	43.6	41.4	39.5	37.7	6.70	46.3	43.5	41.4	39.5	37.8	6.69	0.6%	0.2%	0.0%	0.1%	0.4%	0.2%
	17	42.5	40.5	38.6	36.9	35.3	7.15	42.2	40.3	38.5	36.9	35.5	7.19	0.8%	0.4%	0.2%	0.2%	0.4%	0.6%
	18	39.4	37.6	36.0	34.5	33.1	7.60	39.4	37.5	35.9	34.3	33.1	7.61	0.1%	0.3%	0.2%	0.7%	0.2%	0.1%
	19	36.6	35.1	33.7	32.4	31.1	8.05	36.1	35.0	33.7	32.6	31.0	8.15	1.4%	0.4%	0.0%	0.7%	0.4%	1.3%
	20	34.1	32.8	31.6	30.4	29.3	8.50	33.8	32.9	31.3	30.5	29.3	8.56	1.1%	0.3%	0.7%	0.4%	0.2%	0.6%
	21	31.9	30.7	29.6	28.6	27.6	8.95	31.7	30.7	29.3	28.7	28.0	8.99	0.8%	0.1%	1.1%	0.3%	1.3%	0.5%
	22	29.9	28.8	27.9	26.9	26.1	9.40	29.7	28.6	27.8	26.7	26.4	9.43	0.4%	0.8%	0.1%	0.9%	1.0%	0.4%
	23	28.0	27.1	26.2	25.4	24.7	9.85	27.7	26.9	26.1	25.6	24.5	9.88	1.0%	0.9%	0.4%	0.8%	0.5%	0.3%
	24	26.3	25.5	24.7	24.0	23.4	10.30	25.9	25.4	24.7	23.9	23.5	10.45	1.6%	0.4%	0.1%	0.6%	0.7%	1.4%
	25	24.8	24.1	23.4	22.7	22.1	10.75	24.4	23.9	23.1	22.8	22.0	10.89	1.6%	0.7%	1.0%	0.1%	0.6%	1.3%

Table 7. Comparison of the relocation probability w_{rel} from the analytical approach vs discrete event simulation and the estimation error (mean squared error).

f		Analytical approach						Discrete event simulation						Estimation error (MSE)					
		95%	90%	75%	50%	25%	10%	95%	90%	75%	50%	25%	10%	95%	90%	75%	50%	25%	10%
sh	1	0.00	0.00	0.00	0.00	0.00	0.00	0.00	0.00	0.00	0.00	0.00	0.00	0.000	0.000	0.000	0.000	0.000	0.000
	5	1.88	1.75	1.38	0.77	0.24	0.05	1.89	1.78	1.48	1.01	0.53	0.23	0.000	0.001	0.011	0.060	0.084	0.034
	10	4.25	4.00	3.25	2.00	0.78	0.17	4.25	4.03	3.35	2.23	1.15	0.46	0.000	0.001	0.010	0.050	0.135	0.082
	15	6.63	6.25	5.13	3.25	1.38	0.35	6.64	6.27	5.22	3.48	1.75	0.69	0.000	0.000	0.008	0.055	0.137	0.114
	20	9.00	8.50	7.00	4.50	2.00	0.56	8.99	8.53	7.08	4.74	2.36	0.93	0.000	0.001	0.006	0.059	0.130	0.135
	25	11.38	10.75	8.88	5.75	2.63	0.79	11.41	10.80	8.98	5.97	2.99	1.16	0.001	0.002	0.010	0.050	0.133	0.139
	30	13.75	13.00	10.75	7.00	3.25	1.02	13.73	13.05	10.87	7.24	3.61	1.40	0.000	0.003	0.014	0.056	0.126	0.147
	35	16.13	15.25	12.63	8.25	3.88	1.26	16.14	15.27	12.73	8.51	4.25	1.65	0.000	0.000	0.011	0.068	0.139	0.147
	40	18.50	17.50	14.50	9.50	4.50	1.51	18.51	17.49	14.56	9.71	4.87	1.89	0.000	0.000	0.004	0.042	0.134	0.146
	45	20.88	19.75	16.38	10.75	5.13	1.75	20.93	19.75	16.50	11.05	5.49	2.14	0.003	0.000	0.015	0.088	0.134	0.149
	50	23.25	22.00	18.25	12.00	5.75	2.00	23.38	22.09	18.36	12.30	6.21	2.39	0.017	0.008	0.011	0.093	0.207	0.153
	55	25.63	24.25	20.13	13.25	6.38	2.25	25.79	24.34	20.11	13.56	6.81	2.63	0.027	0.009	0.000	0.099	0.186	0.145
	60	28.00	26.50	22.00	14.50	7.00	2.50	28.03	26.52	22.14	14.80	7.37	2.87	0.001	0.000	0.021	0.090	0.140	0.139
	65	30.38	28.75	23.88	15.75	7.63	2.75	30.46	28.79	23.95	16.08	8.03	3.15	0.007	0.001	0.006	0.111	0.164	0.157
	70	32.75	31.00	25.75	17.00	8.25	3.00	32.78	30.99	25.93	17.40	8.66	3.39	0.001	0.000	0.033	0.161	0.165	0.150
	75	35.13	33.25	27.63	18.25	8.88	3.25	35.10	33.23	27.71	18.58	9.25	3.62	0.001	0.000	0.007	0.111	0.139	0.135
	80	37.50	35.50	29.50	19.50	9.50	3.50	37.72	35.65	29.60	19.86	9.90	3.87	0.050	0.024	0.011	0.130	0.157	0.136
	85	39.88	37.75	31.38	20.75	10.13	3.75	39.86	37.85	31.49	21.11	10.48	4.15	0.000	0.010	0.012	0.132	0.127	0.161
	90	42.25	40.00	33.25	22.00	10.75	4.00	42.26	39.99	33.35	22.32	11.15	4.39	0.000	0.000	0.011	0.101	0.159	0.149
	95	44.63	42.25	35.13	23.25	11.38	4.25	44.67	42.32	35.38	23.56	11.84	4.61	0.002	0.005	0.066	0.095	0.216	0.131
	100	47.00	44.50	37.00	24.50	12.00	4.50	46.93	44.35	37.10	24.77	12.46	4.90	0.005	0.022	0.010	0.075	0.208	0.160
														0.56%	0.42%	1.32%	8.22%	14.37%	12.89%
